# Engineering of In Vitro 3D Capillary Beds by Self-Directed Angiogenic Sprouting

**DOI:** 10.1371/journal.pone.0050582

**Published:** 2012-12-04

**Authors:** Juliana M. Chan, Ioannis K. Zervantonakis, Tharathorn Rimchala, William J. Polacheck, Jordan Whisler, Roger D. Kamm

**Affiliations:** 1 Department of Biological Engineering, Massachusetts Institute of Technology, Cambridge, Massachusetts, United States of America; 2 Molecular Engineering Laboratory, Agency for Science, Technology and Research, Singapore, Singapore; 3 Department of Mechanical Engineering, Massachusetts Institute of Technology, Cambridge, Massachusetts, United States of America; Medical University Innsbruck, Austria

## Abstract

In recent years, microfluidic systems have been used to study fundamental aspects of angiogenesis through the patterning of single-layered, linear or geometric vascular channels. *In vivo*, however, capillaries exist in complex, three-dimensional (3D) networks, and angiogenic sprouting occurs with a degree of unpredictability in all *x*,*y*,*z* planes. The ability to generate capillary beds *in vitro* that can support thick, biological tissues remains a key challenge to the regeneration of vital organs. Here, we report the engineering of 3D capillary beds in an *in vitro* microfluidic platform that is comprised of a biocompatible collagen I gel supported by a mechanical framework of alginate beads. The engineered vessels have patent lumens, form robust ∼1.5 mm capillary networks across the devices, and support the perfusion of 1 µm fluorescent beads through them. In addition, the alginate beads offer a modular method to encapsulate and co-culture cells that either promote angiogenesis or require perfusion for cell viability in engineered tissue constructs. This laboratory-constructed vascular supply may be clinically significant for the engineering of capillary beds and higher order biological tissues in a scalable and modular manner.

## Introduction

The single most pressing challenge in the fields of tissue engineering and regenerative medicine has been the understanding and control of vascularization [1]. *In vivo*, capillary networks deliver oxygen and nutrients to thick (>1–2 mm) tissues which cannot be supported by passive diffusion [2]. Likewise, in pathophysiological conditions, malignant tumors flip an “angiogenic switch,” inducing the formation of abnormal vasculature to support the increased tumor density [3]. There currently exists an enormous clinical demand for engineered soft tissue to treat peripheral artery diseases and myocardial ischemia, and also for the long-term prospect of regenerating vital organs, including the liver, kidney, and heart [4], [5]. For these clinical applications to be possible, a preformed *in vitro* internal vascular supply will be required to maintain the viability of laboratory-engineered tissues and organs with dimensions greater than a few hundred micrometers [4], [5].

Recent advances in cell biology combined with new technologies to manipulate cell behavior have led to a new field where biological systems are miniaturized within microfluidic systems [6], [7]. Researchers have developed microfluidic platforms to study fundamental aspects of angiogenesis and thrombosis *in vitro* [8], [9], such as real-time imaging of anastomoses [10], and the contribution of interstitial pressure to angiogenic sprouting [11]. While these systems are highly informative experimentally, the capillary channels designed in these studies are single-layered vessels that lie at 90-degree angles to each other or in parallel, and do not closely model physiologic capillaries. *In vivo*, both physiologic and pathologic networks respond to angiogenic signals and sprout to form complex, three-dimensional (3D) capillary beds in multiple planes [12]; These capillary beds are multi-layered, undergo repeated branching, and sprout along random paths [13].

Using the same core technology of microfabrication, we built the blueprint for a capillary bed into a biodegradable gel scaffold. Here, the microfluidic platform consists of a collagen matrix embedded with alginate microbeads, and the alginate beads form the 3D architecture and compartmentalization of other cell types that interact with the capillary bed. Human microvascular endothelial cells (hMVECs) seeded in channels were encouraged to sprout with either an exogenous supply of growth factors, or by the encapsulation of highly angiogenic cell lines that secrete growth factors. Capillaries sprouted in 1–2 days and reached invasion depths of ∼1.25 mm within one week. The vascular bed produced using this microfluidic platform was capillary-like in scale, structure, and function. It possessed the “tree-like” structure of native microvasculature, and supported the perfusion of 1 µm diameter particles through patent lumens.

The objective of our study was to design 3D capillary networks that address three key requirements of a vascular construct. First, the vascular construct should allow for the highly organized incorporation of multiple cell types, which ideally should be within ∼100–200 µm of a microvessel [2]. Here, the small dimensions of the alginate beads (152.5±33.4 µm) and the interconnected vascular channels are expected to provide good nutrient and oxygen supply to encapsulated cells. Second, the construct should be fabricated with a scalable strategy in mind, as larger constructs will eventually be necessary to engineer whole organs comprised of densely-packed, metabolically-active cells [14]. In our design, alginate beads encapsulating different cell types can be assembled into any overall size and shape in controlled bead ratios and cell densities, and be functionally connected to a larger caliber vessel to generate a perfused vascular bed. Third, the construct should be designed with a biocompatible scaffold that is U.S. Food and Drug Administration (FDA)-approved for cell-based therapy. Collagen I [15] and alginate [16] are both FDA-approved biomaterials and have been used in various tissue-engineering applications (e.g. AlgiMatrix, Covalon matrix) [17].

**Figure 1:**
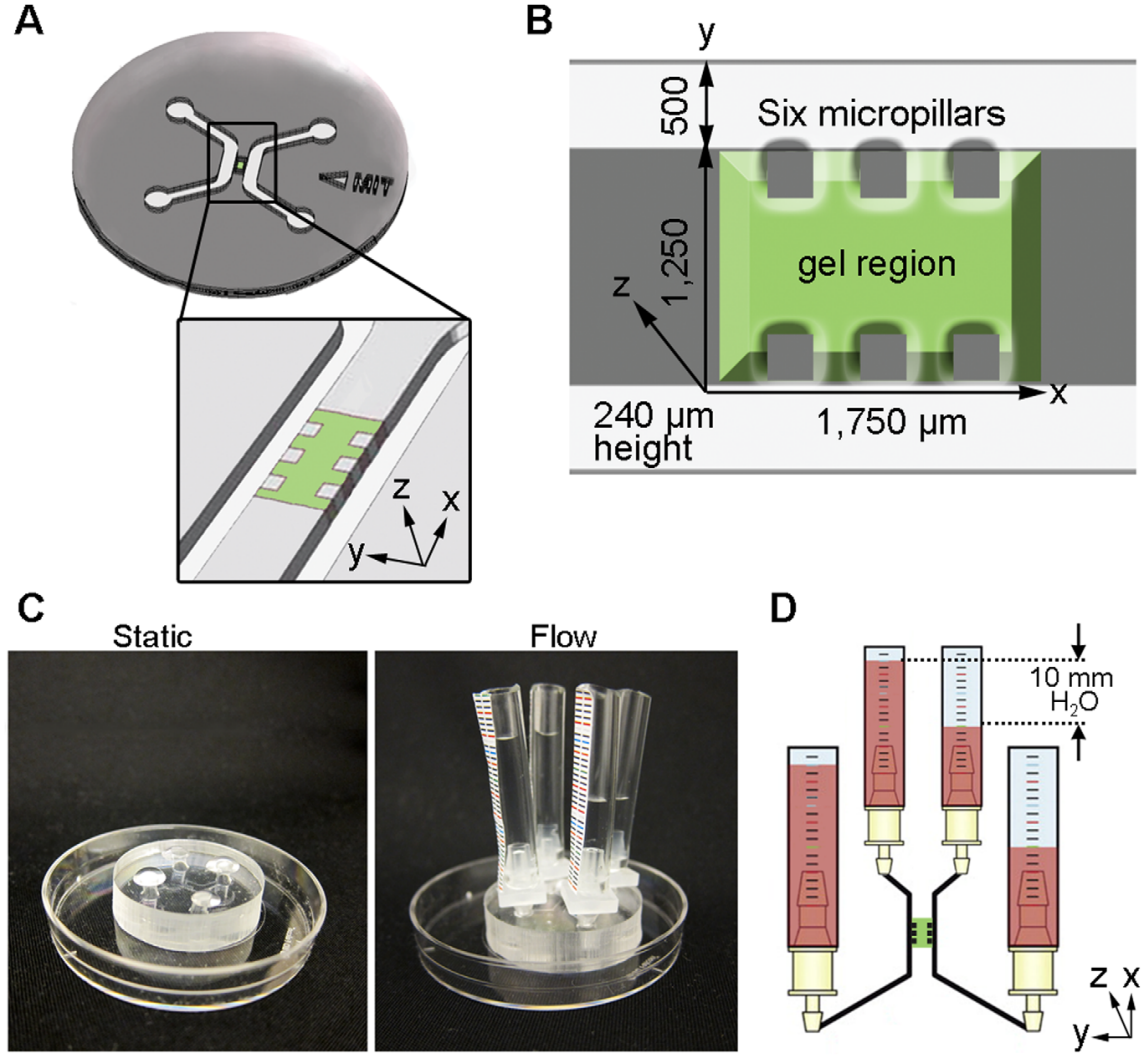
Microfluidic device design and fabrication. (**a**) The microfluidic-based cell culture platform directs vascular growth along an alginate bead scaffold to form a 3D capillary bed. The optically transparent PDMS is bonded to a glass coverslip, and the gel region is flanked by two microfluidic channels with inlet and outlet ports for medium renewal. (**b**) Dimensions of the microfluidic device in µm. The gel region (1,750 µm wide, 1,250 µm long, 240 µm high) is surrounded by six square micropillars (250×250×240 µm) for gel containment. (**c**) Microfluidic devices cultured under static and flow conditions. (**d**) Schematic of 10 mm pressure drop set-up for flow analysis studies.

In summary, we have designed, built, and tested a laboratory-generated capillary bed which shows robust capillary growth with patent lumens, and forms complex, anastomosing capillary networks that support flow. These large, vascularized tissue constructs on the millimeter scale provide the basic architecture to design thicker tissues that go beyond the diffusion limit. The ultimate goal is to scale up the technology into a full, 3D system with the blood volume and cell densities required for a complete organ, and to integrate it into the host circulatory system by anastomoses.

**Figure 2:**
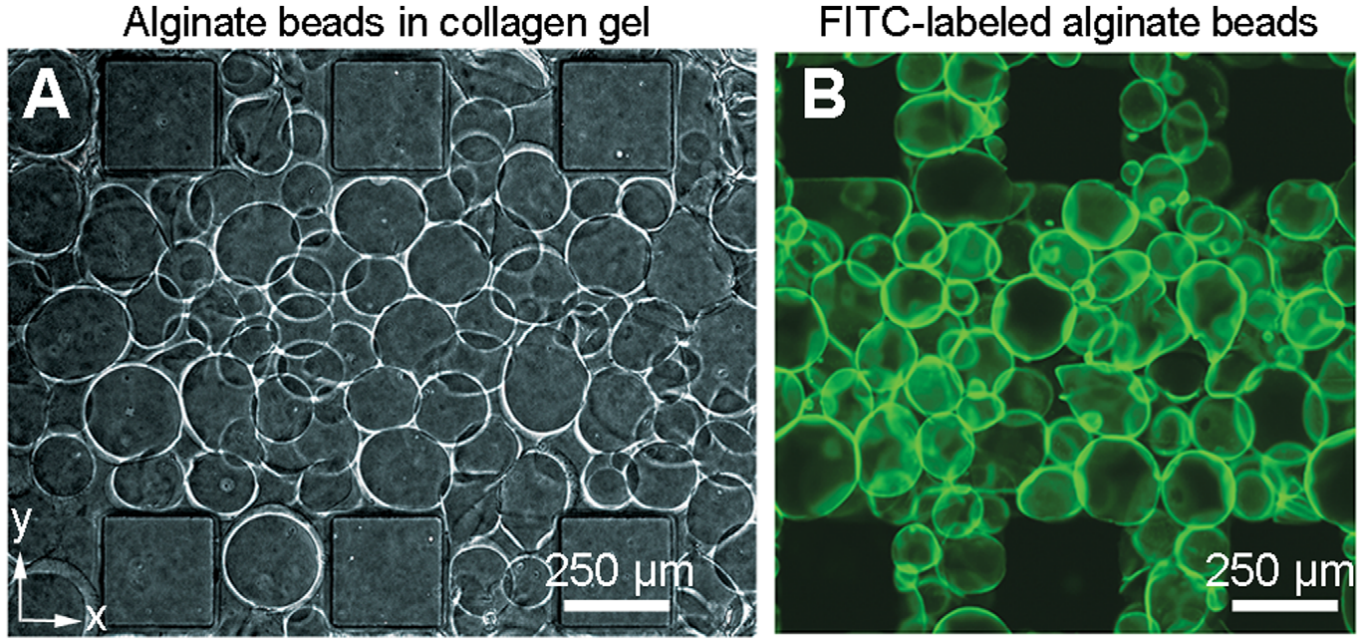
Alginate-collagen hydrogels as a blueprint for 3D capillary bed design. (**a**) Phase contrast image of the alginate-collagen gel region. The alginate beads form 40% of the gel volume. (**b**) Fluorescent image of the alginate-collagen gel region using PLL–FITC coated alginate beads. Scale bars, 250 µm (**a, b**).

## Materials and Methods

### Cell Culture

Human dermal fibroblasts, HDF, and a human fibrosarcoma cell line, HT-1080 (American Type Culture Collection (ATCC), Manassas, VA), were cultured in Dulbecco’s modified Eagle’s medium containing 4.5 g/l glucose (DMEM, Sigma-Aldrich, St Louis, MO), supplemented with L-glutamine, penicillin (100 units/ml), streptomycin (100 µg/ml), and 10% fetal bovine serum (Sigma-Aldrich). hMVECs (Lonza, Williamsport, PA) were cultured in collagen I-treated culture flasks in microvascular endothelial medium-2 (EGM-2MV, Lonza), supplemented with an EGM-2 bullet kit (Lonza). All cells were routinely cultured at 37°C in a humidified incubator containing 5% CO_2_/95% air. HT-1080 and HDF were used up to passage 25 and hMVECs were used at passage 6–8. Medium was changed three times per week and cells were harvested with trypsin-EDTA (Gibco). In devices where more than one cell type was present, both cell types were cultured in hMVEC complete culture medium.

**Figure 3:**
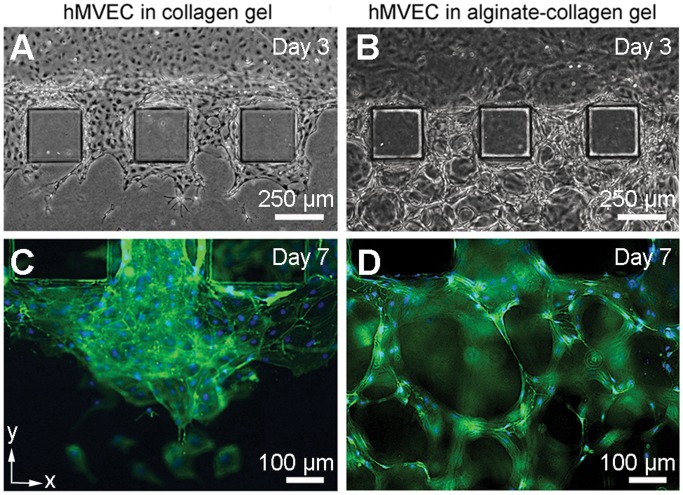
Effects of scaffolding on the 3D properties of the capillary bed. Phase contrast images of hMVECs in (**a**) collagen and (**b**) alginate-collagen gels on Day 3 of hMVEC seeding. Confocal microscopy images of hMVECs in (**c**) collagen and (**d**) alginate-collagen gels on Day 7 of hMVEC seeding stained for vascular endothelial (VE)-cadherin (green) and cell nuclei (blue). Scale bars, 250 µm (**a, b**), 100 µm (**c, d**).

### hMVEC Transfection with Reporter Plasmid

For live-imaging studies, hMVECs were transfected with a pMSCV-puromycin vector containing mCherry gene sequences (MSCV Retroviral Expression System, Clontech Laboratories Inc., Mountain View, CA). Briefly, DNA complexes were prepared by incubating 18 µl of Fugene 6 (Roche Diagnostics, Indianapolis, IN) in 600 µl Opti-MEM reduced serum media (Invitrogen, Grand Island, NY) for 5 min at room temperature, before mixing in 4 µg of plasmid DNA, 0.4 µg of gag/pol DNA, and 2.5 µg of VSV-G DNA (all from Clontech) by gentle flicking. The DNA complexes were added to a 293T packaging cell line (Clontech) cultured in DMEM and 10% fetal bovine serum and gently mixed by swirling. After incubation for 18 h at 37 °C, viruses were harvested from the medium using a 20 ml syringe, and filtered using a 0.45 µm syringe filter. Viruses were aliquoted and frozen at −80°C or used immediately. For hMVEC transfection, the viral supernatant was mixed with 10 ug/ml protamine sulfate (Sigma-Aldrich) from a 3 mg/ml stock solution in PBS. An early hMVEC passage (p 4–5) was incubated with a 1∶1 mix of viral supernatant and complete EGM-2MV medium. The infection was repeated 24 h later to increase the number of hMVECs that were transfected without a positive selection step.

**Figure 4:**
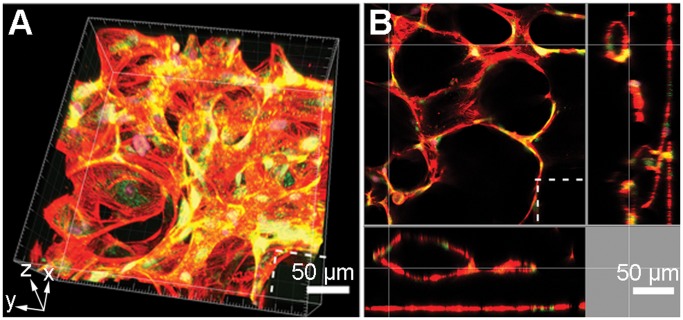
Confocal microscopy reconstruction of the 3D capillary bed. (**a**) Reconstructed *z*-stack at 40× magnification on Day 7 of hMVEC seeding. (**b**) Cross-sectional profile of the *z*-stack shows lumens on both the *x*,*y*-axis planes (VE-cadherin, green; actin, red; cell nuclei, blue). Dotted lines indicate where the PDMS micropillars are located. Scale bars, 50 µm (**a, b**).

### Wafer Fabrication

Microfluidic devices were fabricated using photolithography and soft-lithography techniques [18]. The device design was created in AutoCAD 2009 (Autodesk, San Rafael, CA) and the negative pattern of the device was printed onto a transparency mask with a high-resolution printer. Standard photolithography was employed for wafer processing (Seoulin Biosciences, Seoul, South Korea). Briefly, a SU-8 photoresist was spin-coated on clean silicon wafers at a thickness of 120 µm. Following a pre-bake, the mask was placed over the wafer and the assembly was exposed to ultraviolet (UV) light, causing the photoresist to be photopolymerized where exposed. To achieve a 240 µm *z*-axis, a second layer was made by secondary spin coating and exposure. Next, the wafer was developed, hard-baked, washed with acetone, and dried using compressed air. Wafers were silanized to facilitate removal of replica material, and microfluidic devices were made by replica molding of polydimethylsiloxane, PDMS, (Sylgard 184, Dow Corning, Midland, MI) using a 10∶1 ratio of base to curing agent. After degassing the elastomer mix in a vacuum chamber, the mix was poured onto the wafer and cured in an 80°C oven overnight. Polymerized PDMS devices were peeled off the silicon master, individual devices (30 mm diameter, 10 mm height) were cut from PDMS molds, and inlet and outlet ports were cored down to microfluidic channels using standard 4 mm biopsy punches. Prior to cell culture use, PDMS devices were air-dusted with compressed air, autoclaved at 120°C using a wet cycle (20 min sterilization), and dry cycle (20 min sterilization/15 min dry). Circular glass coverslips were similarly air-dusted with compressed air, and autoclaved at 120°C using a dry cycle (20 min sterilization/15 min dry). PDMS devices and glass coverslips were plasma treated (Harrick Expanded Plasma Cleaner, Harrick Plasma, Ithaca, NY) for 2 min. Surface treatment renders the PDMS surface and coverslip hydrophilic, ensuring a tight bond and preventing leaks.

**Figure 5:**
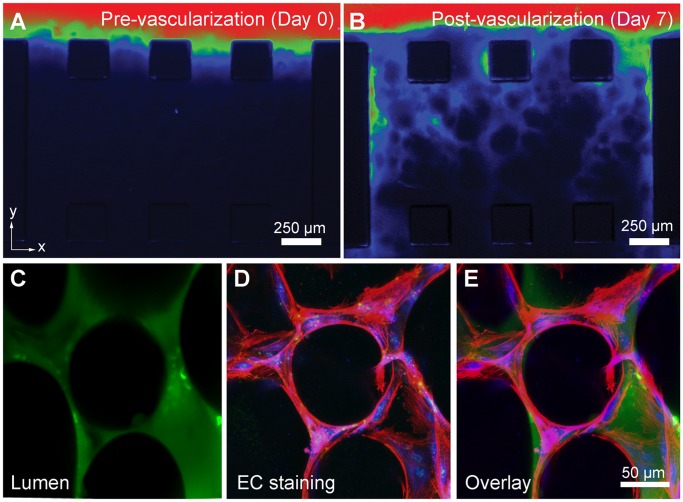
Flow analysis of the 3D capillary bed. (**a**) Representative diffusion patterns of a MW 150,000 fluorescent dextran tracer before vascularization (Day 0) and (**b**) after vascularization (Day 7). (**c**) Confocal images of the FITC–dextran flow patterns (green) in live devices overlaid onto (**d**) images of actin (red) and cell nuclei (blue) staining to give (**e**) a composite image. Scale bars, 250 µm (**a, b**); 50 µm (**c–e**).

### Alginate Bead Formation

Alginate solutions of 4% (w/v) were prepared using low-viscosity alginic acid sodium salt from brown algae (Cat#A2158, Sigma-Aldrich) dissolved in PBS and passed through a 0.45 µm syringe filter (BD Biosciences, Bedford, MA) to remove bacteria and any large clumps of undissolved alginate. If cell encapsulation is required, cells (i.e. HT-1080 fibrosarcomas) were trypsinized, centrifuged (200×*g*, 5 min), and resuspended in complete DMEM medium, before being mixed gently in a 1∶1 ratio with the 4% alginate solution in 1× PBS to give a final 2% alginate solution and 5×10^6^ cells/ml cell density. If cell encapsulation is not required, a 2% alginate solution was used instead. To prepare the alginate beads, the alginate solution (with or without cells) was loaded into a 3 ml syringe and infused using a syringe-pump (Advance Infusion Pump Series 1200, Roboz Surgical Instruments Co., Inc., Gaithersburg, MD) at 25 ml/h through a 30 gauge needle (BD Biosciences) into a focused air-jet stream. The built-in laboratory air supply was connected to Tygon® laboratory tubing and focused using a 200 µL pipette tip at a 90-degree angle to the syringe. The alginate solution was cut using the focused air-jet stream and beads were polymerized by calcium crosslinking in a 15 cm petri-dish containing 100 mM CaCl_2_ solution below the air-jet stream. To isolate ∼150 µm diameter beads from smaller beads and unencapsulated cells, the beads were filtered using 100 µm cell strainers (BD Biosciences) and resuspended in 3–5 ml of DMEM. Cell-encapsulated beads were maintained in a humidified incubator at 37°C and 5% CO_2_. To visualize the alginate beads embedded within the collagen matrix, beads were coated under gentle rocking conditions with 0.4 mg/ml of poly–_L_–lysine-fluorescein isothiocyanate (PLL–FITC, MW 15,000–30,000, P3543 from Sigma-Aldrich) in DMEM for 5 min at room temperature. The beads were centrifuged (200×*g*, 5 min) twice before mixing into the prepolymerized collagen gel.

**Figure 6:**
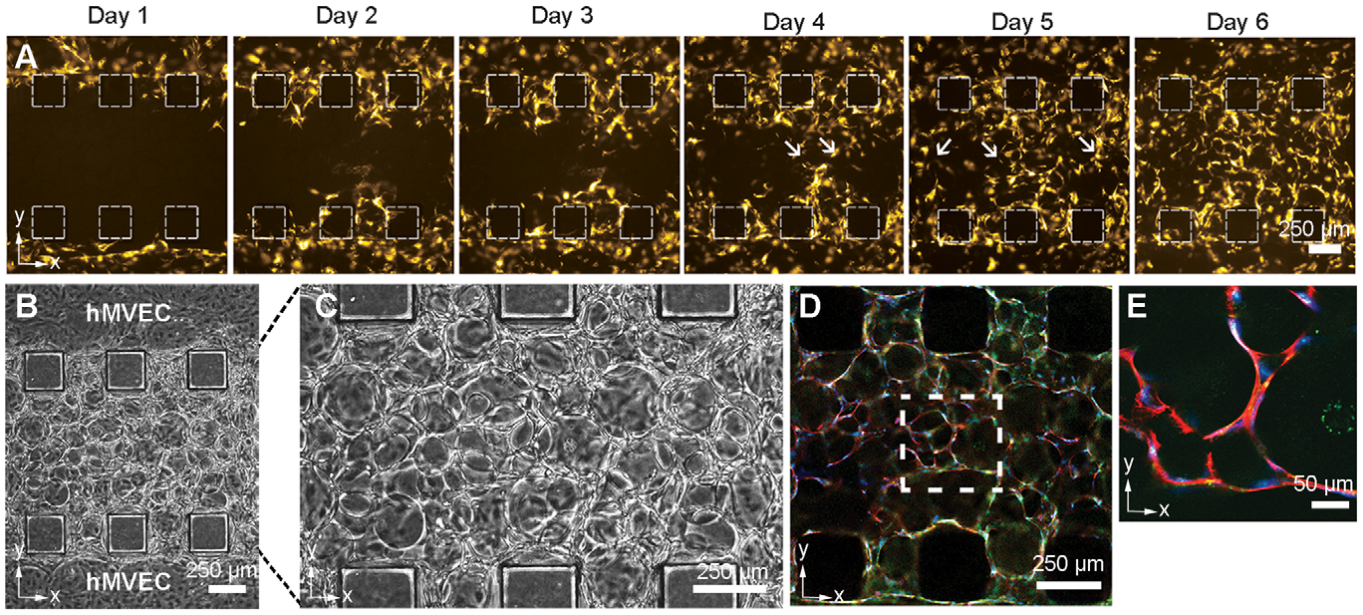
Anastamoses within the 3D capillary bed. (**a**) Fluorescent images of hMVECs undergoing angiogenic sprouting at Day 1–6 after seeding. Arrows indicate the points of contact where vessels anastamose and form lumens. (**b**) Phase contrast images of 3D microvascular networks in alginate-collagen gels at 4**×** magnification and (**c**) 20**×** magnification. (**d**) High resolution confocal images taken at Day 6 show specific sites of anastomoses, and dotted boxes correspond to images at (**e**) higher magnification (VE-cadherin, green; actin, red; cell nuclei, blue). Scale bars, 250 µm (**a–d**), 50 µm (**e**).

### Gel Filling of Device

Sterile PDMS devices and glass coverslips were filled with gels within 30–60 min of surface plasma treatment. A prepolymer collagen solution containing alginate beads was prepared on ice by combining: rat tail collagen type I (BD Biosciences), 10× PBS solution, 0.5 M NaOH, and 40% (v/v) alginate beads in suspension, to produce a final collagen concentration of 2.5 mg/ml at pH 7.4, excluding the 40% volume of alginate beads. The alginate-collagen solution was carefully mixed 10–20 times without introducing any air bubbles. The solution was carefully microinjected using a 10 µl pipette into the gel region until the six micropillars surrounding the gel region were no longer visible (final gel volume ∼0.525 µl). After microinjection, the PDMS devices were immediately bonded to glass coverslips. The devices were placed in a humidified container with the glass slide facing downwards, and allowed to polymerize for 30 min in a 37°C humidified incubator with 5% CO_2_. Finally, the microfluidic channels were filled with cell culture medium.

**Figure 7:**
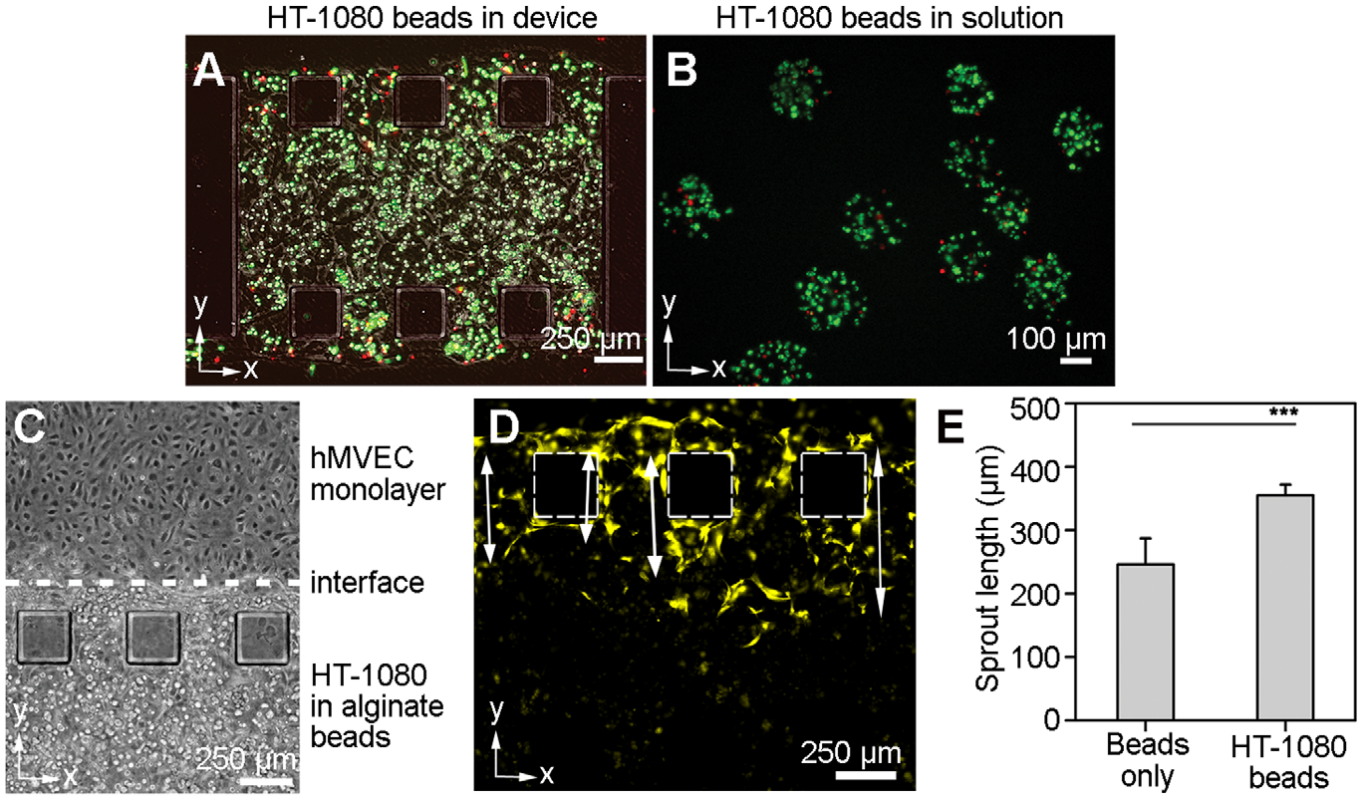
Coculture interactions within the 3D capillary bed. (**a**) Fluorescent microscopy image of a microfluidic device loaded with HT-1080 alginate beads with live/dead staining (24 h after gel filling). Live cells are stained green, and dead cells are stained red. (**b**) Fluorescent microscopy image of HT-1080 alginate beads in solution with live/dead staining at Day 7. (**c**) Phase contrast image taken on Day 1 of coculture shows the interface between the hMVEC monolayer in the channel, and HT-1080 alginate beads within the gel region. (**d**) hMVEC sprout lengths were measured as indicated by the arrows in the image. (**e**) hMVEC sprout lengths at 72 h after seeding in devices with alginate beads and HT-1080 alginate beads (***, p<0.005). Scale bars, 100 µm (**b**); 250 µm (**a, c, d**).

### hMVEC Seeding

24 h later after gel filling, hMVECs were added at a density of 2×10^6^ cells/ml to one channel of the device. The hMVECs were allowed to attach for 60 min by flipping the devices sideways such that the cells formed a monolayer across the gel. After 60 min of seeding, devices were flipped back and channels were washed with fresh culture medium. Each port was filled with an additional 60 µl culture medium to prevent evaporation. The next day, additional 50 ng/ml VEGF_165_ (Peprotech, Rocky Hill, NJ) was added to the medium to stimulate angiogenesis and proliferation. If angiogenesis is not needed, the EGM-2 bullet kit (Lonza) supplied VEGF_165_ would be sufficient for cell maintenance. hMVEC medium was replaced daily.

### Diffusion Studies

To characterize the transport of growth factors secreted by cells, diffusion experiments were performed using different molecular weight dextrans (Cascade Blue-conjugated, MW 10,000; FITC-conjugated, MW 40,000; and Texas Red-conjugated, MW 70,000; all from Invitrogen) mixed with culture medium at a final concentration of 15 µg/ml. The fluorescent solution was added to the condition channel while PBS was added to the other channel. A series of fluorescent images of the gel region was acquired at 30 min intervals for 2 h using a Nikon TE300 fluorescence microscope (Nikon Instruments Inc., Melville, NY). Image processing of time-lapse fluorescent images was performed using a custom written code in MATLAB (Mathworks, Boston, MA, USA) [19]. Briefly, a vertical line was drawn through the gel region and the fluorescence intensity was mapped to a concentration profile by subtracting the background intensity value and normalizing to the maximum intensity value in the condition channel. The diffusion coefficients for the different molecular weight dextrans **(Figure S1)** were quantified by fitting the analytical solution of the one-dimensional transient diffusion to the measured fluorescent intensity data.

### Immunocytochemistry and Imaging

Tissue constructs were imaged 7–9 days post-assembly by both fluorescence and confocal microscopy. The devices were washed twice in PBS and fixed with 4% paraformaldehyde (Electron Micoscopy Sciences, Hatfield, PA) for 20 min at room temperature. After two washes in PBS, the cells were permeabilized with 0.2% Triton X-100 for 10 min at room temperature. After three washes in PBS, the devices were blocked with 5% goat serum in 0.5% BSA/PBS solution for 1 h at room temperature. hMVECs were stained with rabbit anti-vascular endothelial (VE)-cadherin antibody (ALX-210-232-C100, 100 µg/ml stock solution in ultrapure water, Enzo Life Sciences International, Plymouth Meeting, PA) at 1∶100 dilution in 0.5% BSA/PBS solution overnight at 4°C. The next day, the devices were washed five times with 0.5% BSA/PBS solution and incubated with Alexa Fluor 488-conjugated anti-rabbit IgG (P2771, 1 mg/ml, Invitrogen) at 1∶200 dilution for 2 h at room temperature. After three washes in 0.5% BSA/PBS, actin filaments and cell nuclei were labeled with rhodamine phalloidin (R415, 300 U, Invitrogen) at 1∶100 dilution, and 4′,6-diamidino-2-phenylindole, DAPI (D8417, 1 mg/ml stock solution in ultrapure water, Sigma-Aldrich) at 1∶1000 dilution for 1 h at room temperature, followed by three washes in PBS. For 2D fluorescence imaging, images were obtained using a Nikon TE300 fluorescence microscope described earlier. For 3D confocal imaging, a *z*-axis series of fluorescence images was obtained using a Zeiss 510n confocal laser scanning microscope (Carl Zeiss MicroImaging, Thornwood, NY, USA). 1 µm thick ∼150–200 optical serial *z*-axis sections were obtained and reconstructed in 3D using Imaris 7.1.1 software (Bitplane Inc., Saint Paul, MN).

### Cell Viability Assays

Cell viability assays in microfluidic devices: Cells in alginate beads were labeled live/dead using a LIVE/DEAD Reduced Biohazard Cell Viability Kit (Invitrogen) according to the manufacturer’s instructions. Briefly, the devices were incubated in 240 µl of Hank’s buffered salt solution containing the live/dead stain for 30 min at room temperature in the dark. The distribution of green (live) and red (dead) cells was visualized using a TE300 Nikon fluorescence microscope at 4× magnification.

Cell viability assays in solution: The trypan blue exclusion method was used to quantify the number of live cells in the alginate beads. 500 µl of cell encapsulated alginate beads were dissolved in 55 mM sodium citrate (Sigma-Aldrich) and placed on a hemocytometer. Cells that had trypan blue exclusion were counted as viable cells, and percentage cell viability was determined from *n* = 5 samples, ∼30 cells per field of view.

### hMVEC Sprouting Coculture Studies with HT-1080 Alginate Beads

HT-1080 cells were encapsulated in alginate beads, and mixed with collagen I gels using the standard gel filling procedure. 24 h after gel filling, hMVECs were seeded at a density of 2×10^6^ cells/ml in one channel of the device, and allowed to attach for 60 min by flipping the device perpendicularly. Subsequently, the cell medium was replaced with a basal microvascular endothelial medium-2 (EGM-2MV) that was not supplemented with additional growth factors. The medium was changed at 24 h intervals. At 72 h after hMVEC seeding, the lengths of four of the longest sprouts per device were measured and averaged among *n* = 7 devices per condition using ImageJ software (NIH).

### Statistical Analysis

Error bars represent standard deviation, with *n* ≥ 3 for all experiments. Statistical significance was determined using one-way ANOVA followed by Tukey’s Multiple Comparison Test (*P*<0.05). All statistical analysis was performed using Origin 7.0 data analysis software (OriginLab, Northampton, MA).

## Results

### Microfluidic Device Fabrication and Assembly

The alginate-collagen hydrogel device consists of molded silicone rubber (polydimethylsiloxane, PDMS) bonded to a glass coverslip, with a central gel region flanked by two microfluidic channels supplied by four inlet/outlet ports [20]. **Fig. 1a** shows a schematic of the microfluidic device and **Fig. 1b** provides its dimensions. The microfluidic devices can support both static and flow conditions (**Fig. 1c**), and flow studies were carried out using a pressure drop as shown in the schematic (**Fig. 1d**).

To assemble the device, a composite hydrogel consisting of alginate beads (40% gel volume) in 2.5 mg/ml collagen I (final collagen concentration) was filled into the gel region by microinjection (volume of gel region = 0.525 µl) to give 119±10 alginate beads per gel region (*n* = 4 devices) (**Fig. 2a**). During the gel filling process, the gel is constrained within the gel region by six micropillars (250×250×240 µm). The average size distribution of the alginate beads after size filtration was 152.5±33.4 µm using ImageJ analysis of ∼30 beads per section (*n* = 4 devices), a size range both suitable for gel filling and the diffusion of nutrients and oxygen to encapsulated cells. To visualize the alginate beads embedded within the collagen matrix, the beads were coated with poly–_L_–lysine-fluorescein isothiocyanate (PLL–FITC, MW 15,000–30,000) before mixing into the prepolymerized collagen gel. A representative *z*-section of the gel region taken by confocal microscopy is shown in **Fig. 2b**.

### Analysis of Diffusion Properties Across Alginate-collagen Gels

An initial concern was that the collagen would not polymerize completely when 40% of the gel volume was alginate. In **Fig. S1**, we collected images of diffusion gradients produced with FITC–dextran of different sizes (MW 10,000 to 70,000) across these gels. Barring the uneven fluorescence intensity profile across the alginate-collagen gels, both the collagen-only and collagen-alginate gels produced comparable diffusion gradients at 2 h **(Fig. S1a, b)**. The diffusion coefficient of the 40 kDa dextran across the alginate-collagen gel at D_ALG–COL_ = 2.09×10^−11^ m^2^/s is lower than for the 2.5 mg/ml collagen I gel at D_COL_ = 2.54×10^−11^ m^2^/s, as dextran diffusion is hindered by the smaller diffusion coefficient of alginate **(Fig. S1c)**. A complete set of diffusion profiles and coefficients can be found in **Fig. S1**. Hence, the alginate-collagen gel maintains the original barrier function of collagen, with added features of scaffolding and spatially-restricted cocultures.

### Characteristics of the 3D Microcapillary Bed Produced with an Alginate-collagen Scaffold

Preliminary experiments were carried out to explore conditions that led to angiogenic sprouting. Highly angiogenic conditions were achieved with hMVEC medium containing angiogenic factors (e.g. FGF-b, IGF-1, EGF), and supplemented with 50 ng/ml recombinant human vascular endothelial growth factor isoform (VEGF_165_). The collagen concentration used in the study was also important for controlling angiogenic sprouting. 2 mg/ml alginate-collagen gels (0.2%) resulted in rapid hMVEC sprouting; in contrast, 3 mg/ml (0.3%) alginate-collagen gels resulted in limited hMVEC sprouting. Hence, 2.5 mg/ml (0.25%) collagen gels polymerized at pH 7.4 were used in all experiments.

Examination of phase contrast and immunofluorescent images of collagen-only gels (**Fig. 3a, c**) against alginate-collagen gels (**Fig. 3b, d**) showed a marked dependence of the vascular 3D features on the architecture provided by the beads. The speed of hMVEC sprouting was also improved in the presence of the alginate beads comparing representative images taken on Day 3 and Day 7 (**Fig. 3**). hMVEC angiogenic sprouting in collagen-only gels was inadequate along the *z*-axis, defined as the distance from the bottom of the device to which sprouts penetrate divided by the total height of the gel region, expressed as a percentage (Δ, sprouting along *z*-axis), Δ_COL_ = 24.4±8.83% vs. Δ_ALG–COL_ = 65.5±9.42% (p<0.005, *n* = 8 devices). In collagen-only gels, the hMVECs preferentially migrated along the glass coverslip at the bottom of the device, resulting in approximately 75% of the gel region remaining nonvascularized. In alginate-collagen gels, however, the capillary bed extended along the *z*-axis, covering more than 65% of the gel region. Our measurements showed that the median vessel diameter was 15 µm (mean: 18.5±9.5 µm, *n* = 40), taken midway between bifurcation points from phase contrast images of the devices. Only vessels that were in focus and distinct from other vessels were selected for measurement. It was, however, also noted that the vessels diameter at the bifurcation points were larger than midway between them, and that the vessels may not all be patent. The vessels were slightly larger than *in vivo* capillary diameters which range from 5–10 µm, but otherwise in the same range. Hence, it was evident that vascularization along the *z*-axis (Δ) and complex features of the 3D capillary bed were a result of the scaffolding provided by the alginate beads.

Immature microvessels tend to be thin and form slit-like intracellular lumens completely enclosed by a single endothelial cell (seamless capillaries) [21]. Functional microvessels, however, show larger endothelial tube formations with complete lumens lined by multiple endothelial cells. Confocal images taken at 40× magnification were reconstructed to give a volumetric image in 3D (**Fig. 4a**). Together with *x*- and *y*-axis cross-sections (**Fig. 4b**), the images showed that the capillaries had complete, multicellular lumens which stained for actin (red) and vascular endothelial (VE)-cadherin (green) at endothelial cell junctions. The microvessels were also reconstructed from 156 µm *z*-section frames taken at high magnification (40×) in **Video S1**.

### Flow Studies in the 3D Capillary Bed

Within alginate-collagen gels, hMVECs seeded along one channel rapidly sprout into fully formed capillaries, lined with multiple endothelial cells consistent with patent lumens, and reach invasion depths of ∼ 1.25 mm that protrude into the other channel after 6–8 days.

To visualize the flow patterns generated within the 3D capillary beds, devices were subject to pressure differences of a 10 mm water column (100 Pa) with cell culture medium. High molecular weight FITC–dextran (MW 150,000) was added to one channel and fluorescent images were taken 5 min after dextran had perfused through the network. A comparison of dextran profiles before (Day 0) (**Fig. 5a**) and after vascularization (Day 7) (**Fig. 5b**) shows the formation of a complex capillary bed that supports flow.

A representative confocal image of the flow patterns generated from FITC–dextran perfusion in devices (**Fig. 5c**) was overlaid onto images of the same network subsequently fixed and stained for actin (red) and cell nuclei (blue) (**Fig. 5d**), to give a composite image that shows the flow occurred through the immunostained endothelial cells (**Fig. 5e**). The fraction of patent vessels was calculated using confocal images similar to **Fig. 5e** at 10× magnification. Using images at different *z*-axis planes, taking care not to select the same sprout twice, we identified 46 individual sprouts that had been stained for actin and cell nuclei. All identified vessels were ≤50 µm in diameter midway between vessel bifurcation points. Next, we observed FITC–dextran confocal images and correlated each vessel with dextran perfusion. A total of 80.4% of vessels (37 patent, 9 non-patent; *n* = 46) were observed to be patent. The true fraction of patent vessels may be higher as the fluorescence intensity in smaller vessels may be below detection limits.

Functional perfusion of the 3D capillary bed was demonstrated using 1 µm diameter fluorescent microparticles. Fluorescence time-lapse imaging of the gel region at 0.5 s intervals reveals the perfusion of 1 µm diameter particles through well-defined and interconnected microvessels. After concluding the flow experiments, the same device was fixed and immunostained for VE-cadherin (green) and cellular nuclei (blue), and merged with time-lapse images (**Video S2**).

### Examination of Anastamoses in the 3D Capillary Bed

Microvessels that develop within this microfluidic device recapitulate a number of important angiogenic stages *in vivo*: cell sprouting, migration, proliferation, lumen formation, branching, and anastomosis [22]. Cellular sprouting processes were dynamic, with the tip cells changing direction and branching from previous sprouts (SI Video 3).

To visualize the anastamosing vessels, hMVECs were transfected with mCherry retroviral reporter plasmids. Unlike earlier experiments where hMVECs were seeded in one channel of the device, here mCherry-fluorescent hMVECs were seeded in both channels with a 12 h interval between seedings. Time-lapse images over five days show the invasion of capillary networks from both channels (**Fig. 6a**). The vessels develop an extensive 3D capillary bed around the beads, with arrows indicating points where cells contacted another vessel. Consistent with the hypothesis of multiple anastomoses are visually confirmed positions where sprout tip cells meet on Day 4 and 5 (**Fig. S2**). It was, however, technically challenging to dynamically track individual sprouts in complex 3D networks in real-time. For example, confocal images in **Fig. 6d** and **Fig. 6e** could not be taken on Day 4 or 5 as it is a terminal experiment with fixation and staining, and so the images had to be taken after the sprouts that were tracked visually had already anastamosed.

After 4–5 days, a complex 3D capillary bed of interconnected capillary vessels was formed, with representative images taken at 4× magnification (**Fig. 6b**) and at 20× magnification (**Fig. 6c**). High resolution confocal images taken of the immunostained networks (**Fig. 6d**) allow us to identify specific anastomoses on Day 6 (**Fig. 6e**).

### Functional Properties of the 3D Capillary Bed in Coculture with Alginate-encapsulated Cells

Finally, we investigated if the hMVECs were able to achieve cross-talk with cells encapsulated in alginate beads, either by responding to growth factor release, or by providing a supply of nutrients. In this experiment, we tested whether the hMVECs would respond to vascular growth signals secreted by a cocultured HT-1080 human fibrosarcoma cell line. We first quantified the potential of HT-1080s to induce angiogenesis via protein secretion using an angiogenesis antibody array (**Fig. S3**). By comparing HT-1080s against primary human dermal fibroblasts (**Fig. S3a**), we showed that the HT-1080s proliferated much more rapidly (**Fig. S3b**), and secreted VEGF at 2.5-fold levels over fibroblasts after normalizing for cell number (**Fig. S3c, f**). Fibroblasts secreted angiogenin [23] at four-fold levels over HT-1080s, a factor important during physiologic angiogenesis (**Fig. S3d, f**). In contrast, HT-1080s secreted placental growth factor (PIGF) [24], a potent tumor angiogenic factor not detected with fibroblasts (**Fig. S3e, f**).

Next, HT-1080 cells were encapsulated in alginate beads at a density of 5×10^6^ cells/ml in 2% (w/v) alginate, and microinjected as an alginate-collagen gel. The number of cells per device was determined to be 1,318±217 cells/device (*n* = 8 devices). HT-1080 cell viability was quantified using live/dead staining in devices (**Fig. 7a**). Cells were randomly distributed and most appeared to be alive (green), while those along the gel boundary appeared dead (red). Cell death at the gel-air interface may have occurred during gel polymerization at 37°C. In addition to live/dead staining in devices, daily viability measurements were taken from alginate-encapsulated cells in cell medium using a trypan blue exclusion assay (**Fig. 7b**). Over one week, the viability of the encapsulated cells decreased gradually from 84.4±6.1% on Day 1 to 74.4±5.1% on Day 7 (*n* = 5, ∼30 cells per section).

HT-1080 encapsulated alginate beads were microinjected into the device, and hMVECs were seeded in one channel after a 24 h interval, with the interface shown in **Fig. 7c**. The cell culture medium did not contain added growth factors (e.g. FGF-b, IGF-1, EGF, VEGF), so as to quantify angiogenic stimulation from only the HT-1080 cell population. At 72 h after hMVEC seeding, four of the longest sprouts were measured per device, and averaged among *n* = 7 devices per condition (**Fig. 7d**). The longest sprout distance for the beads-only devices was 246.10±40.85 µm, whereas the longest sprout distance for the HT-1080 bead devices was 354.78±16.88 µm (p<0.005, *n* = 7 devices) (**Fig. 7e**). As the alginate beads also act as a physical barrier to prevent direct cell-cell contact between the encapsulated cells and the capillary bed, the contribution of HT-1080 to angiogenic sprouting was likely through the secretion of growth factors such as VEGF and PIGF.

## Discussion

The approach described here to engineer *in vitro* vascularized tissues uses a modular scaffold comprised of two FDA-approved biomaterials. To encourage endothelial sprouting, we delivered chemical angiogenic stimuli using two methods: (a) stimulating vessel growth into the scaffold with soluble angiogenic factors such as exogenous VEGF_165_ [25]; and (b) stimulating vessel growth into the scaffold by encapsulating cells that secrete angiogenic factors [26].

There have been a number of existing strategies from our lab and others towards the design of microvascular networks *in vitro*. In principle, most of the published studies fall into two broad design groups: the first uses a biocompatible hydrogel such as collagen I and fibrin which is subsequently invaded by endothelial sprouts [10], [11], [27]; while the second uses a sacrificial template or mold such as poly(lactic-co-glycolic)acid (PLGA) [28], poly(glycerol sebacate) (PGS) [29], or carbohydrate glass [30] to delineate the channels that are later seeded with endothelial cells.

Whereas the goal of the first type of platform is typically to study fundamental aspects of angiogenesis *in vitro* using methods such as confocal microscopy and real-time imaging, these systems are generally single-layered and are not intended for scale-up tissue engineering applications. In the second type of platform, the diameters of the vessels produced by sacrificial templates or molds fall in the 50–150 µm range and larger. Vessels on that scale are more reflective of small arterioles, versus capillaries whose diameters are in the order of 5–10 µm *in vivo*. Small arterioles designed using a template or mold are by default also pre-patterned, meaning that they do not undergo the angiogenic sprouting processes that give rise to both physiologic and pathologic capillaries.

The strength of our system lies in our ability to control the overall blueprint of the capillary bed while still maintaining a high degree of self-directed vessel sprouting. Not only do the alginate beads provide the 3D architecture of the capillary bed, the vessels also sprouted more readily when the beads were present. This observation we believe is linked to the variable stiffness of the matrix microenvironment, with the alginate beads having a higher modulus than the collagen. The morphology of the vessels produced using our approach also mimics physiologic capillaries, such as their ∼15 µm vessel diameters, multicellular lumens, and anastomosing networks. Further, we were able to demonstrate the vascularization of a large fraction of the *z*-axis (Δ_ALG–COL_ = 65.5±9.42%) with relative ease, such that most encapsulated cells are within 100–200 µm of a capillary vessel, as would be necessary in an *in vitro* engineered soft tissue.

Although ∼15 µm diameter vessels were plentiful and formed the majority of capillaries, we note that these may not necessarily all represent functional and patent vessels. Perfusion experiments conducted with MW 150,000 fluorescent dextran in **Fig. 5** and 1 µm diameter fluorescent particles in **Video S2** showed the preference for dextran and particle flow through larger vessels in the 50–100 µm range. This is to be expected, since the larger vessels will have the lower flow resistance. Despite the shunting effect observed in larger vessels, fluorescent dextran still perfused a large fraction of the ∼15 µm range capillaries (**Fig. 5b**) and 1 µm diameter particles also perfused smaller capillaries (**Video S2**).

While other studies have shown the feasibility of coculture within vascular networks, our approach has an added advantage of modularity. It is possible to engineer cocultures or even tricultures by mixing alginate beads encapsulating different cell types. We demonstrated the feasibility of coculture using HT-1080 fibrosarcomas encapsulated in alginate beads, as these cells are highly angiogenic and secrete a variety of angiogenic factors, including PIGF, a potent angiogenic factor that promotes pathologic angiogenesis [24]. Future studies may use cell types that are beneficial in physiologic angiogenesis, such as fibroblasts [31], smooth muscle cells, and pericytes. Mural cell types may easily be incorporated into the devices, and the cells may cooperate with newly formed capillaries to regulate vessel permeability and maturation [32].

For these 3D capillary beds to be clinically useful, it will be necessary to show viability and function beyond the two weeks achieved in this study, especially in the presence of physiological fluid flow. Future studies will focus on long-term vessel maturation using growth factors such as angiopoietin-1 (Ang-1) [33] and sphingosine-1-phosphate (S-1-P) [34], together with flow. Previous studies suggest that the combination of these factors is likely to stabilize vascular networks and reduce endothelial permeability in the absence of stromal cells [35], [36].

### Conclusion

Functional 3D tissues in microscale devices designed with multicellular and multimaterial compositions will soon form the core of next generation tissue engineering approaches. In conclusion, we envision that this vascular construct, delivered with supportive cell populations in biocompatible scaffolds, may have clinical applications when vascularized in the appropriate tissue or organ-like shape, and connected to the vascular supply of the host by anastamoses.

## Supporting Information

Figure S1 **Analysis of diffusion profiles in collagen and alginate-collagen gels. (a)** Diffusion profiles of fluorescent tracers across collagen and **(b)** alginate-collagen gels, taken at 30 min intervals for 2 h as described in the **Methods S1**. Fluorescent tracers used were DAPI–dextran (MW 10,000) (blue), FITC–dextran (MW 40,000) (green), and Texas red–dextran (MW 70,000) (red). Temporal evolution of normalized fluorescent intensity for collagen-only (0% v/v alginate beads, blue open circles) and alginate-collagen (40% v/v alginate beads, red open circles) devices using **(c)** 40 kDa and **(d)** 70 kDa dextrans. The normalized intensity was calculated as the ratio of the value at the midpoint between the two channels to the maximum value in the condition channel [19]. Diffusion coefficients of the gels were estimated by fitting the analytical solution (solid lines, blue and red for the 0% v/v and 40% v/v gels respectively) to the measured intensities (open circles). For collagen-only gels, diffusion coefficients for 40 and 70 kDa dextrans were: D_COL,40_ = 2.54×10^−11^ m^2^/s and D_COL,70_ = 2.35××10^−11^ m^2^/s, while for 40% alginate-collagen gels values were lower (hindered diffusion): D_ALG–COL,40_ = 2.09×10^−11^ m^2^/s and D_ALG–COL,70_ = 1.93×10^−11^ m^2^/s. Scale bar, 250 µm **(a,b)**.
(TIF)

Figure S2 **Analysis of anastomoses on Day 4 and 5 of hMVEC sprouting.** Phase contrast images taken on **(a)** Day 4 and **(c)** Day 5 show that the sprouts are invading the collagen-alginate gels. Identical phase contrast images taken on **(b)** Day 4 and **(d)** Day 5 are reproduced with additional markings to show the pattern of the sprouts (red) and points of contact (yellow circles). Scale bar, 250 µm **(a-d)**.
(TIF)

Figure S3 **Cellular protein expression. (a)** Phase contrast images of cells from a HT-1080 human fibrosarcoma cell line and human dermal fibroblasts (HDF). **(b)** Cellular proliferation of HT-1080 versus HDF cells over 72 h. **(c)** Protein expression levels of VEGF, **(d)** angiogenin, and **(e)** PIGF, tested by an angiogenesis antibody array as described in the **Methods S1**. **(f)** Growth factor secretion levels at 24 h normalized to cell number. Scale bar, 10 µm **(a)**.
(TIF)

Video S1 **3D reconstruction of a microvascular network** ***z*** **-stack.** Microvascular networks were imaged by confocal microscopy at 40× magnification. Devices were fixed and immunofluorescently labeled to detect vascular networks: VE-cadherin on cell membranes (anti-VE-cadherin antibody, green), cytoplasmic actin (rhodamine-phalloidin, red), and cell nuclei (DAPI, blue). 156 *z*-section frames were taken at 1 µm intervals. The video is playing at 15 frames/s. Scale bar, 50 µm.
(AVI)

Video S2 **Time-lapse imaging of flow through 3D capillary bed.** The flow of fluorescent 1 µm diameter beads through the 3D capillary bed was imaged using time-lapse fluorescence microscopy at 10**×** magnification. After imaging, devices were fixed and immunofluorescently labeled to detect vascular networks: VE-cadherin on cell membranes (anti-VE-cadherin antibody, green), and cell nuclei (DAPI, blue) (actin staining in the red channel is not shown here for image clarity). Images were taken at 500 ms intervals, and the video is playing at 6 frames/s. Scale bar, 100 µm.
(AVI)

Video S3 **Time-lapse imaging of the initial hMVEC sprout formation.** hMVEC sprout formation is a dynamic process, and here a single hMVEC sprout probes the 3D gel matrix by sending its filopodia in two directions from the confluent monolayer interface, before retracting the sprout. Images were taken using phase contrast microscopy at 10**×** magnification in 50 min intervals, and the video is playing at 1 frame/s. Total movie duration: 8 h 25 min (a total of 11 frames). Scale bar, 20 µm.
(AVI)

Methods S1 **Supporting Methods.**
(DOC)
