# Supplementary figures and images for: Engineering of In Vitro 3D Capillary Beds by Self-Directed Angiogenic Sprouting
Source: PLoS One. 2012 Dec 4;7(12):e50582. doi: 10.1371/journal.pone.0050582 (PMC3514279; doi:10.1371/journal.pone.0050582)

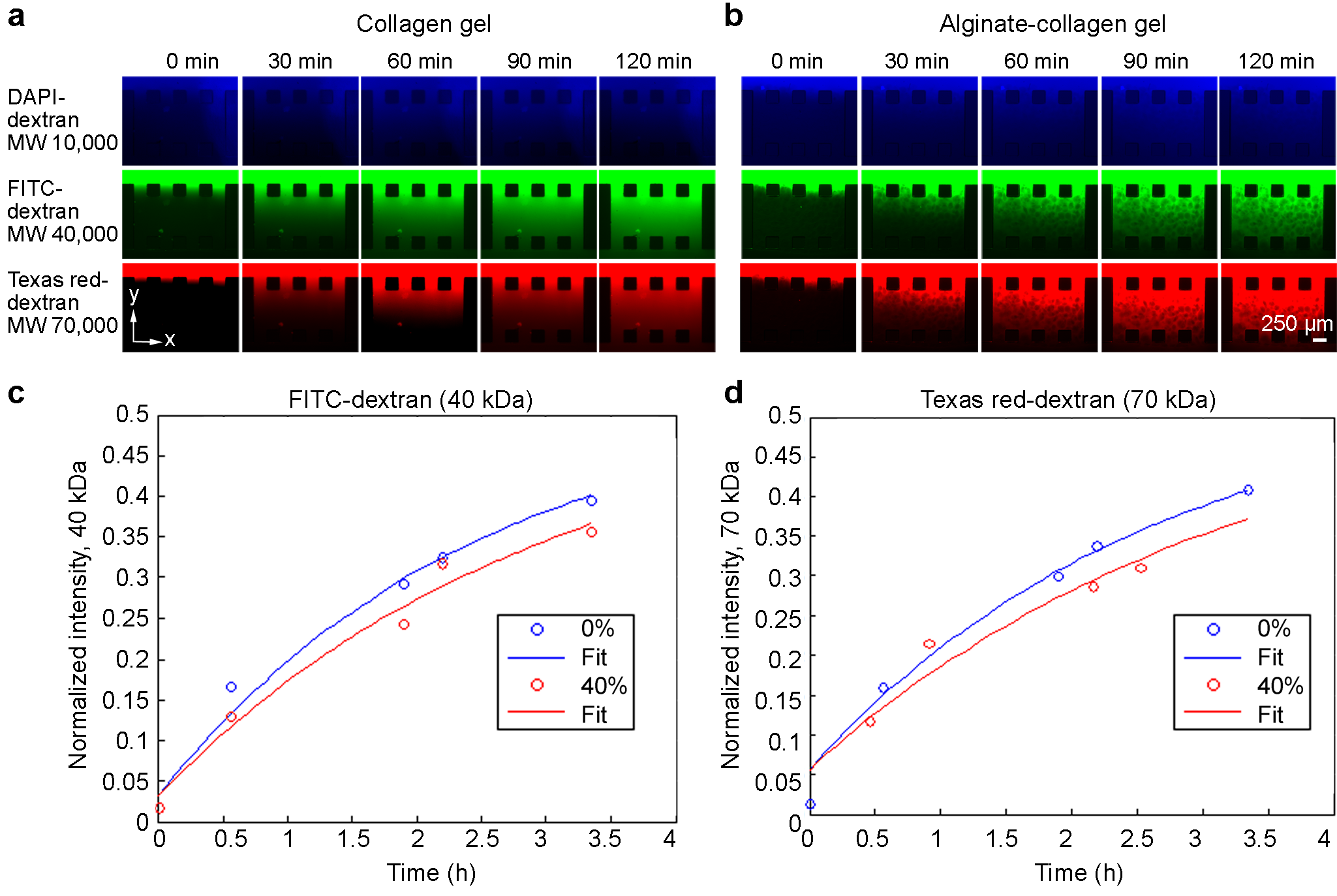

Supplement: Figure S1 — Analysis of diffusion profiles in collagen and alginate-collagen gels. (a) Diffusion profiles of fluorescent tracers across collagen and (b) alginate-collagen gels, taken at 30 min intervals for 2 h as described in the Methods S1. Fluorescent tracers used were DAPI–dextran (MW 10,000) (blue), FITC–dextran (MW 40,000) (green), and Texas red–dextran (MW 70,000) (red). Temporal evolution of normalized fluorescent intensity for collagen-only (0% v/v alginate beads, blue open circles) and alginate-collagen (40% v/v alginate beads, red open circles) devices using (c) 40 kDa and (d) 70 kDa dextrans. The normalized intensity was calculated as the ratio of the value at the midpoint between the two channels to the maximum value in the condition channel [19]. Diffusion coefficients of the gels were estimated by fitting the analytical solution (solid lines, blue and red for the 0% v/v and 40% v/v gels respectively) to the measured intensities (open circles). For collagen-only gels, diffusion coefficients for 40 and 70 kDa dextrans were: DCOL,40 = 2.54×10^−11^ m2/s and DCOL,70 = 2.35××10−11 m2/s, while for 40% alginate-collagen gels values were lower (hindered diffusion): DALG–COL,40 = 2.09×10^−11^ m2/s and DALG–COL,70 = 1.93×10^−11^ m2/s. Scale bar, 250 µm (a,b). (TIF) [file pone.0050582.s001.tif]

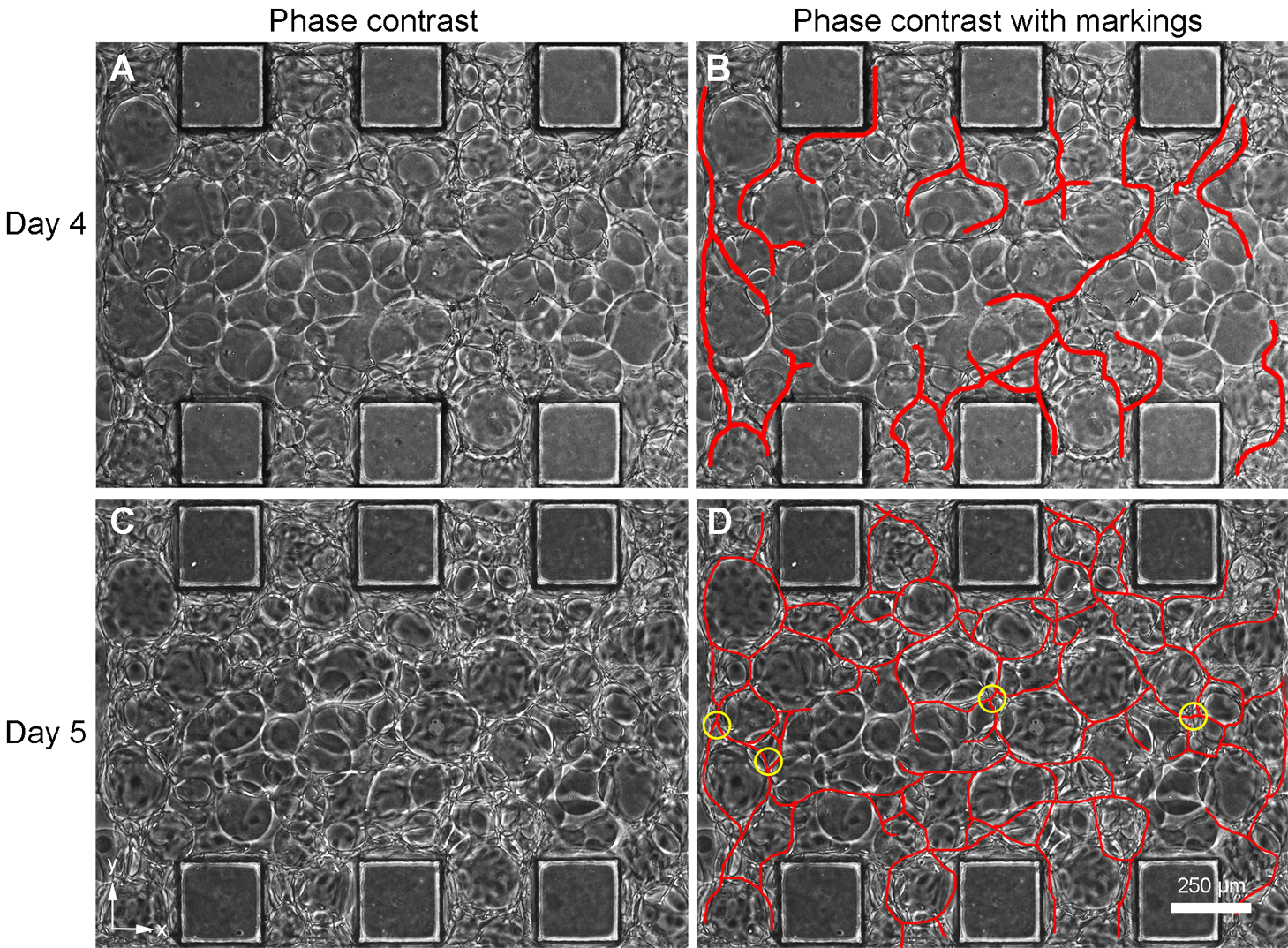

Supplement: Figure S2 — Analysis of anastomoses on Day 4 and 5 of hMVEC sprouting. Phase contrast images taken on (a) Day 4 and (c) Day 5 show that the sprouts are invading the collagen-alginate gels. Identical phase contrast images taken on (b) Day 4 and (d) Day 5 are reproduced with additional markings to show the pattern of the sprouts (red) and points of contact (yellow circles). Scale bar, 250 µm (a-d). (TIF) [file pone.0050582.s002.tif]

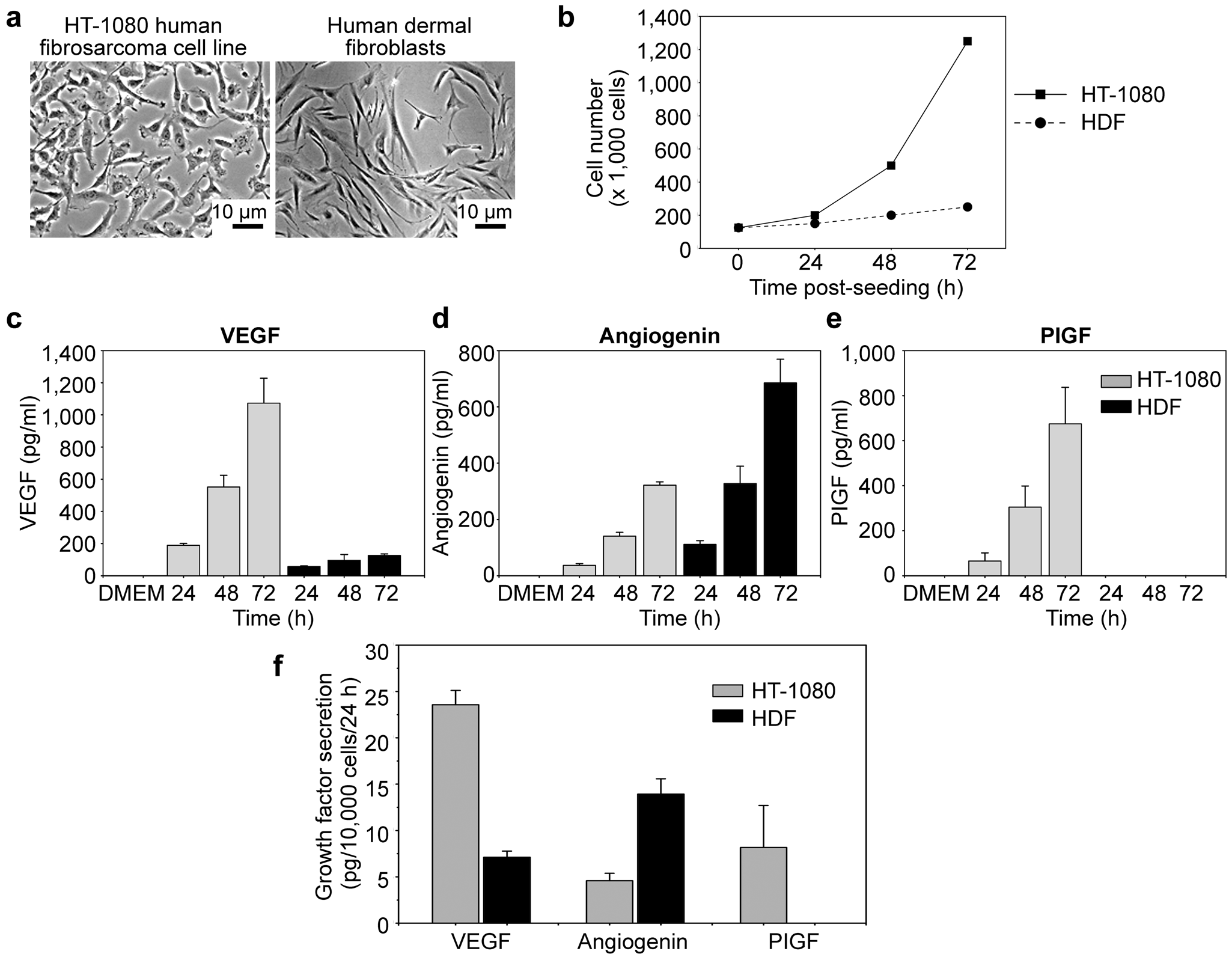

Supplement: Figure S3 — Cellular protein expression. (a) Phase contrast images of cells from a HT-1080 human fibrosarcoma cell line and human dermal fibroblasts (HDF). (b) Cellular proliferation of HT-1080 versus HDF cells over 72 h. (c) Protein expression levels of VEGF, (d) angiogenin, and (e) PIGF, tested by an angiogenesis antibody array as described in the Methods S1. (f) Growth factor secretion levels at 24 h normalized to cell number. Scale bar, 10 µm (a). (TIF) [file pone.0050582.s003.tif]
