# Supplementary material for: Engineering of In Vitro 3D Capillary Beds by Self-Directed Angiogenic Sprouting
Source: PLoS One. 2012 Dec 4;7(12):e50582. doi: 10.1371/journal.pone.0050582 (PMC3514279; doi:10.1371/journal.pone.0050582)
Supplement: Methods S1 — Supporting Methods. (DOC) [file pone.0050582.s007.doc]

**Supporting Methods**

Cell culture

Human dermal fibroblasts, HDF, and a human fibrosarcoma cell line, HT-1080 (American Type Culture Collection (ATCC), Manassas, VA), were cultured in Dulbecco’s modified Eagle’s medium containing 4.5 g/l glucose (DMEM, Sigma-Aldrich, St Louis, MO), supplemented with L-glutamine, penicillin (100 units/ml), streptomycin (100 μg/ml), and 10 % fetal bovine serum (Sigma-Aldrich). hMVECs (Lonza, Williamsport, PA) were cultured in collagen I-treated culture flasks in microvascular endothelial medium-2 (EGM-2MV, Lonza), supplemented with an EGM-2 bullet kit (Lonza). All cells were routinely cultured at 37 °C in a humidified incubator containing 5 % CO_2_/95 % air. HT-1080 and HDF were used up to passage 25 and hMVECs were used at passage 6-8. Medium was changed three times per week and cells were harvested with trypsin-EDTA (Gibco). In devices where more than one cell type was present, both cell types were cultured in hMVEC complete culture medium.

Diffusion studies

To characterize the transport of growth factors secreted by cells, diffusion experiments were performed using different molecular weight dextrans (Cascade Blue-conjugated, MW 10,000; FITC-conjugated, MW 40,000; and Texas Red-conjugated, MW 70,000; all from Invitrogen) mixed with culture medium at a final concentration of 15 μg/ml. The fluorescent solution was added to the condition channel while PBS was added to the other channel. A series of fluorescent images of the gel region was acquired at 30 min intervals for 2 h using a Nikon TE300 fluorescence microscope (Nikon Instruments Inc., Melville, NY). Image processing of time-lapse fluorescent images was performed using a custom written code in MATLAB (Mathworks, Boston, MA, USA)[19]. Briefly, a vertical line was drawn through the gel region and the fluorescence intensity was mapped to a concentration profile by subtracting the background intensity value and normalizing to the maximum intensity value in the condition channel. The diffusion coefficients for the different molecular weight dextrans **(Fig. S1)** were quantified by fitting the analytical solution of the one-dimensional transient diffusion to the measured fluorescent intensity data.

Vascular perfusion studies

To characterize the perfusion of a fully formed 3D capillary bed, 1 µm diameter fluorescent beads were flowed through the capillary bed from one channel using a pressure drop of ~10 mm H_2_O. A series of fluorescent images of the gel region was acquired at 500 ms intervals using a Nikon TE300 fluorescence microscope (Nikon Instruments Inc., Melville, NY) at 10× magnification. After imaging, the devices were fixed and immunofluorescently-labeled to detect vascular channels, and the images were overlaid with the time-lapse video (**SI Video 2**).

Acquisition of conditioned medium and growth factor quantification

Conditioned medium from HT-1080 and HDF cultures were obtained by maintaining cells (seeding density of 2.5 x 10^4^ cells/cm^2^) in six-well plates with 3 ml of cell culture medium. At 24, 48, and 72 h, conditioned medium was removed, centrifuged to remove any potential debris, filtered, and stored frozen at -20 °C until assayed. A human VEGF ELISA kit (Peprotech) was used to quantify the amount of VEGF released into the conditioned medium by absorbance measurements using a VMax Kinetic ELISA microplate reader (Molecular Devices, Sunnyvale, CA). The same samples were also tested for expression of other angiogenic factors with an angiogenesis antibody array kit (Quantibody Human Angiogenesis Array 1, Raybiotech Inc., **Norcross, GA**) using a GenePix 4200 AL microarray laser scanner and Genepix Pro software (Molecular Devices). All procedures were based on the manufacturer’s instructions. DMEM with 10 % FBS was used as a negative control for all procedures. At each time point, the number of cells per well was counted using a hemocytometer to quantify the amount of growth factor secreted per 10,000 cells (pg/10,000 cells/24 h).
